# DNA barcodes of birds from northern Colombia

**DOI:** 10.3897/BDJ.9.e64842

**Published:** 2021-05-21

**Authors:** Paulo Cesar Pulgarín-R, Martha Olivera-Angel, Luisa Ortíz, Duván Nanclares, Sara Velásquez-Restrepo, Juan Fernando Díaz-Nieto

**Affiliations:** 1 Facultad de Ciencias y Biotecnología, Universidad CES, Medellín, Colombia Facultad de Ciencias y Biotecnología, Universidad CES Medellín Colombia; 2 Biogénesis, Facultad de Ciencias Agrarias, Universidad de Antioquia, Cl. 73 #73A-79, Medellín, Colombia Biogénesis, Facultad de Ciencias Agrarias, Universidad de Antioquia, Cl. 73 #73A-79 Medellín Colombia; 3 Grupo Biodiversidad, Evolución y Conservación (BEC), Departamento de Ciencias Biológicas, Escuela de Ciencias, Universidad EAFIT, Carrera 49 No. 7 sur-50, Medellín, Colombia Grupo Biodiversidad, Evolución y Conservación (BEC), Departamento de Ciencias Biológicas, Escuela de Ciencias, Universidad EAFIT, Carrera 49 No. 7 sur-50 Medellín Colombia

**Keywords:** aves, lowland tropical forest, mtDNA, northern Colombia

## Abstract

DNA barcode datasets are a useful tool for conservation and aid in taxonomic identification, particularly in megadiverse tropical countries seeking to document and describe its biota, which is dropping at an alarming rate during recent decades. Here we report the barcodes for several low elevation bird species from northern Colombia with the goal to provide tools for species identification in this region of South America. We blood-sampled birds in a lowland tropical forest with various degrees of intervention using standard 3 × 12 m mist-nets. We extracted DNA and sequenced the COI barcode gene using standard primers and laboratory methods. We obtained 26 COI sequences from 18 species, 10 families and three orders and found that barcodes largely matched (but not always) phenotypic identification (> 90%) and they also facilitated the identification of several challenging passerine species. Despite our reduced sampling, our study represents the first attempt to document COI barcodes for birds (from blood samples) in this part of Colombia, which fills a considerable gap of sampling in this part of South America.

## Introduction

DNA barcode reference libraries are a useful tool for conservation and aid in taxonomic identification (Gonzalez et al. 2009, Waugh 2007) for many biological groups (Hebert et al. 2003). Megadiverse countries, such as Colombia, are desperately in need of documenting and describing its biota, which is declining at an alarming rate during recent decades (Shaw et al. 2013), with emphasis on the putative cryptic diversity present in tropical areas (Crawford et al. 2012, Lohman et al. 2010, Stefan et al. 2018). Despite efforts to encourage sequence data collection and sharing through local and global initiatives (e.g. Barcode Life Data System, BOLD), most taxa are under-represented for barcodes (de Kerdrel et al. 2020, Gaytán et al. 2020, Ko et al. 2013). Naturally, although barcodes are particularly useful for advancing on the recognition of unknown diversity (in groups where alpha taxonomy is still developing or for highly diverse groups where many species remain to be described), it is also extremely useful for species identification in groups with better resolution in their taxonomy (Collins and Cruickshank 2012, Hebert and Gregory 2005, Hebert et al. 2003). Birds are one of the most well-known groups in terms of their taxonomy and systematics (Jarvis et al. 2014); nonetheless, new species are being described almost every year, particularly in the Neotropics (Avendaño et al. 2015, Avendaño et al. 2017) and some challenges still remain in the identification of species groups with very little phenotypic differentiation (Lara et al. 2012, Tavares et al. 2011, Cadena et al. 2016). Consequently, birds are an excellent group for implementing DNA barcoding for both species-identification and species-recognition purposes.

Barcode studies in Neotropical birds are on the increase, particularly in Brazil and Argentina, where studies have focused on testing species limits and biogeographic patterns (Chaves et al. 2015, Kerr et al. 2009, Tavares et al. 2011, Vilaca et al. 2006). Despite that progress, a huge gap in information remains to be filled in northern South America, where very few studies have been completed (but see Mendoza et al. 2016). Here, we report the barcodes for 19 low elevation bird species from northern Colombia with the goal to provide tools for species identification and add to the existing gap in animal COI data in this part of South America.

## Materials and Methods

### Sample collection and processing

**Figure 1.**
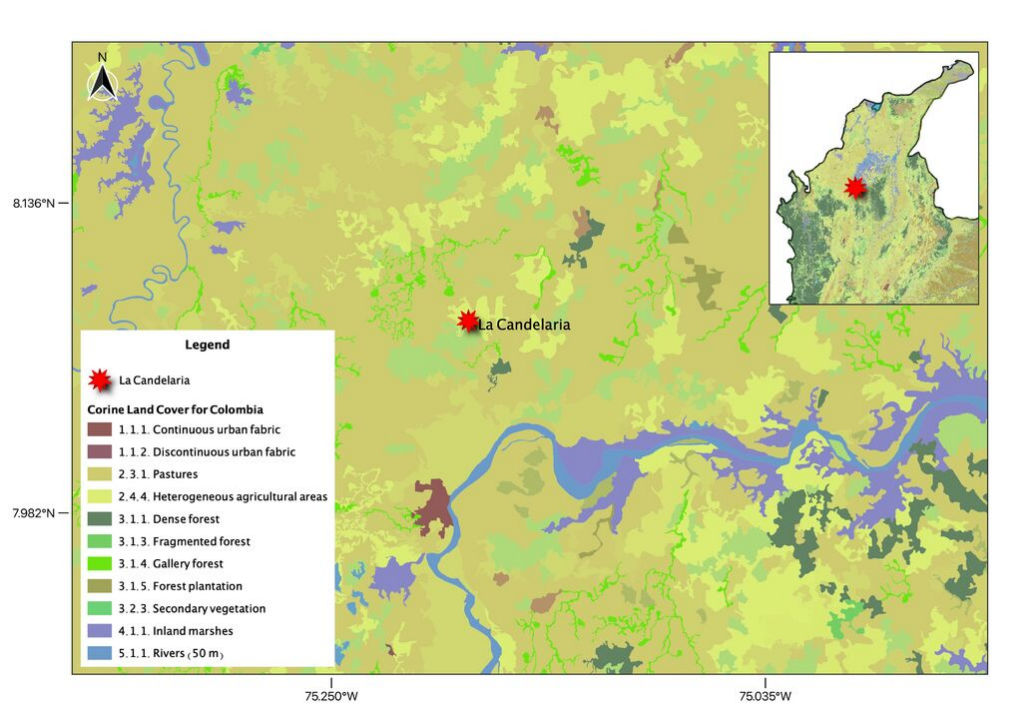
Study area in the lowlands of northern Colombia.

We sampled birds at “Hacienda Universidad de Antioquia”, in the Municipality of Caucasia, Department of Antioquia, Colombia (8.003143 N, -75.400716 W; 70 m a.s.l., Fig. 1), from the 26th to 29th of October 2017. The landscape at the study site is composed of remnants of low land riparian forests, immersed in a matrix of pastures, secondary vegetation, shrubs and small streams (IDEAM 2012). Birds were caught at forest edges and in open areas between forest fragments using standard 3 x 12 m mist-nets and were blood-sampled from the brachial vein, using small gauge needles and non-heparinised capillary tubes (Pulgarín-R et al. 2018). All captured birds were processed, identified using field guides (Hilty et al. 1986, Donegan and McMullan 2014) and finally released in place. Blood samples were stored in 90-95% ethanol and kept at room temperature (Pulgarín-R et al. 2018).

### Laboratory Procedures

We extracted total DNA from blood using the PureLink Genomic DNA Mini Kit (Invitrogen) according to the manufacturer’s specifications. For blood samples, 20 µl of Proteinase K, 20 µl of RNase and 200 µl of PureLink® Genomic Lysis/Binding buffer were added during the digestion phase. Later, each sample was transferred to a spin column and two washes were performed with Wash Buffer 1 and Wash Buffer 2 to perform a final elusion, dividing the total volume into two consecutive sets of 50 µl with Elution Buffer.

For molecular typing, we targeted the Cytochrome c oxidase subunit 1 (COI) barcode region, using the primer combination from Ivanova et al. (2007) with the unique difference that all primers were M13-tailed to facilitate the sequencing process (Table 1). PCR amplifications were performed in 35 µl reactions that contained: 2 mM of MgCl_2_, 1 × of buffer PCR 10 × with KCl, 0.2 mM of each dNTP, 0.14 µl of each primer cocktail, 1U of Taq DNA Polymerase (Fermentas) and 100 ng of DNA template. Thermal cycling conditions involved an initial denaturation at 95°C for 2 min followed by a single stage of 28 cycles that included denaturation at 95°C for 30 s, annealing at 52°C for 40 s, extension at 72°C for 1 min and a final 10 min extension at 72°C. PCR products were visualised on a 1.5% agarose gel, using a MiniBIS Pro-DNR Bio Imaging Systems. All amplification products were purified using Shrimp Alkaline Phosphatase and sent to Macrogen (Seoul, Korea) to be sequenced on an ABI PRISM 3100 Genetic Analyzer (Applied Biosystems, Carlsbad, CA, USA).

### Data analysis

Sequences were edited, assembled and examined with reference to translated amino-acid sequences, using Geneious PRO 6.1.6. Nucleotide-sequences and complementary information were deposited in BOLD (www.barcodinglife.org) with the accession number dataset CANDE030-20 to CANDE055-20. For an initial sequence quality check and provisionary identification, all assembled sequences were searched in the National Centre for Biotechnology Information (NCBI) database through BLAST (http://BLAST.ncbi.nlm.nih.gov/BLAST.cgi), using the Geneious Pro 6.1.6 match tool. We used the top-matching hit having the highest (> 98%) maximal percent identity score as criteria for successful conspecific/congeneric identification. After the initial BLAST-based identification on the NCBI database, we used the Animal Identification (COI) tool from the BOLD Identification System (IDS), using the Species Level Barcode Records database. For all our sequences, we recovered the species identification, closest matching BIN (Table 2) and a Neighbour-Joining topology, using Kimura-2-Parameter (K2P) substitution model as implemented in the BOLD portal (Suppl. material 1).

## Results

We obtained 26 COI sequences from 18 species, 10 families and three orders and, when analysed by BOLD, the species were grouped into 18 existing BINS (access numbers in Table 2). Most bird species were residents, but four species (*Catharus minimus*, *Catharus ustulatus*, *Myiodynastes luteiventris* and *Parkesia noveboracensis*), were boreal migrants (Ayerbe 2018, Avendaño et al. 2015, Avendaño et al. 2017). For all species, sequence lengths varied from 642 to 702 bp (Table 2). Since most bird species are under-sampled for DNA barcodes (Mendoza et al. 2016) in this part of the tropics, our report represents an important contribution to expand the geographic sampling (for COI sequences) of several species in South America and it also includes the first sequences for Colombia for the following species: *Cantorchilus leucotis*, *Chaetura brachyura*, *Galbula ruficauda*, *Myiodynastes luteiventris*, *Myiozetetes cayanensis*, *Tolmomyias sulphurescens* and *Xiphorhynchus susurrans*.

**Figure 2.**
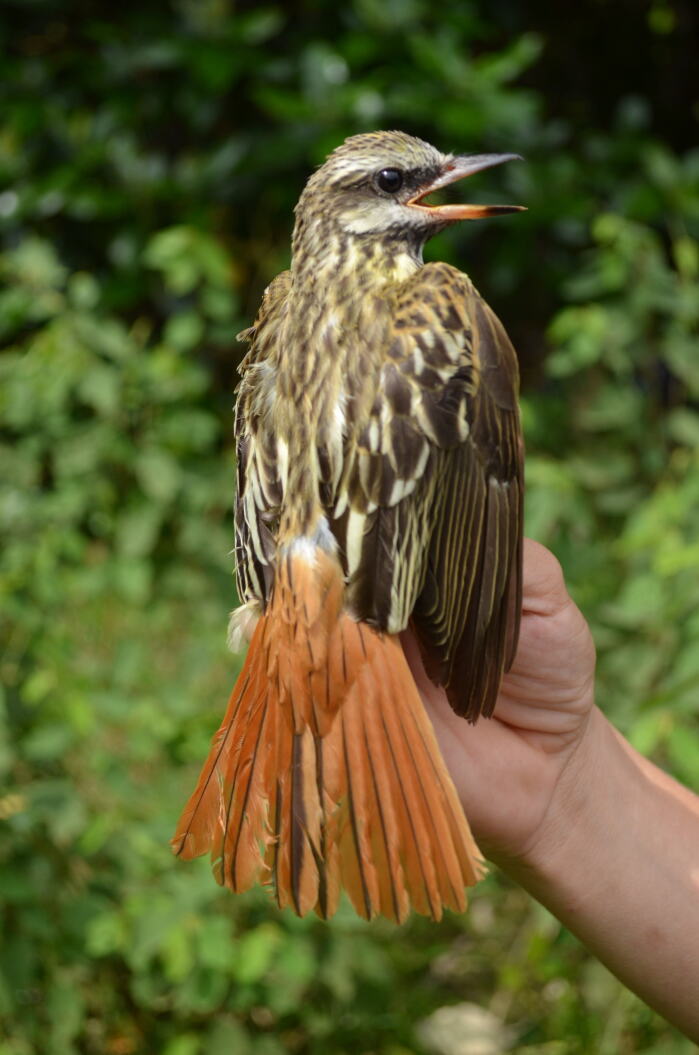
*Myiodynastes luteiventris* (but see Discussion), a boreal migrant, was initially identified in the field as *Myiodynastes maculatus* and was subsequently re-identified with the help of its COI barcode (BOLD ID).

**Figure 3.**
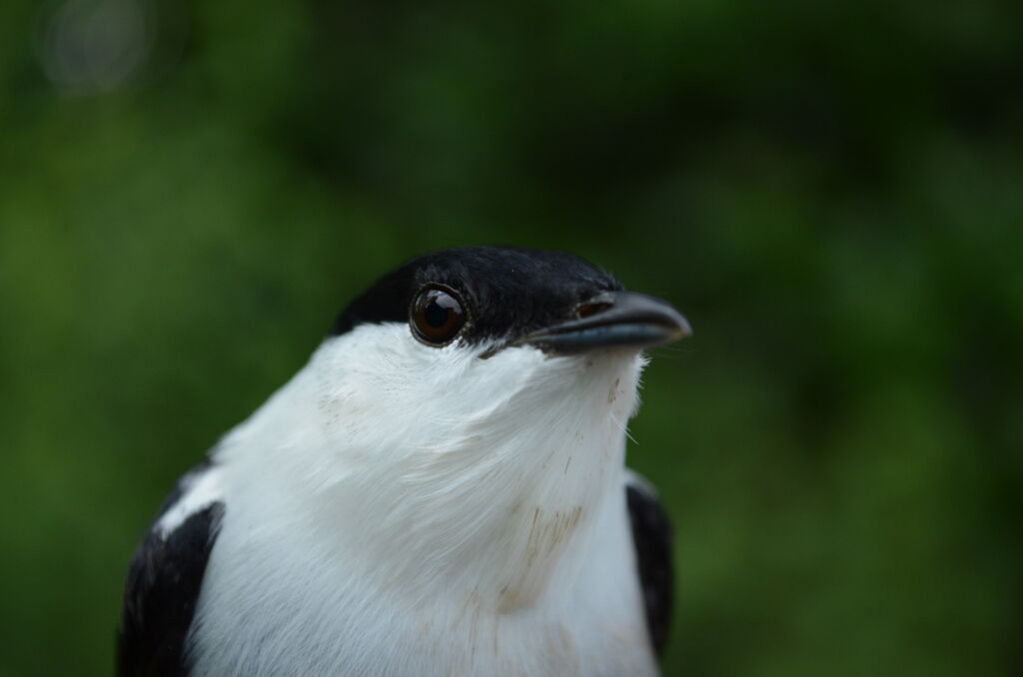
*Manacus manacus* was identified as a different manakin species according to BOLD.

**Figure 4.**
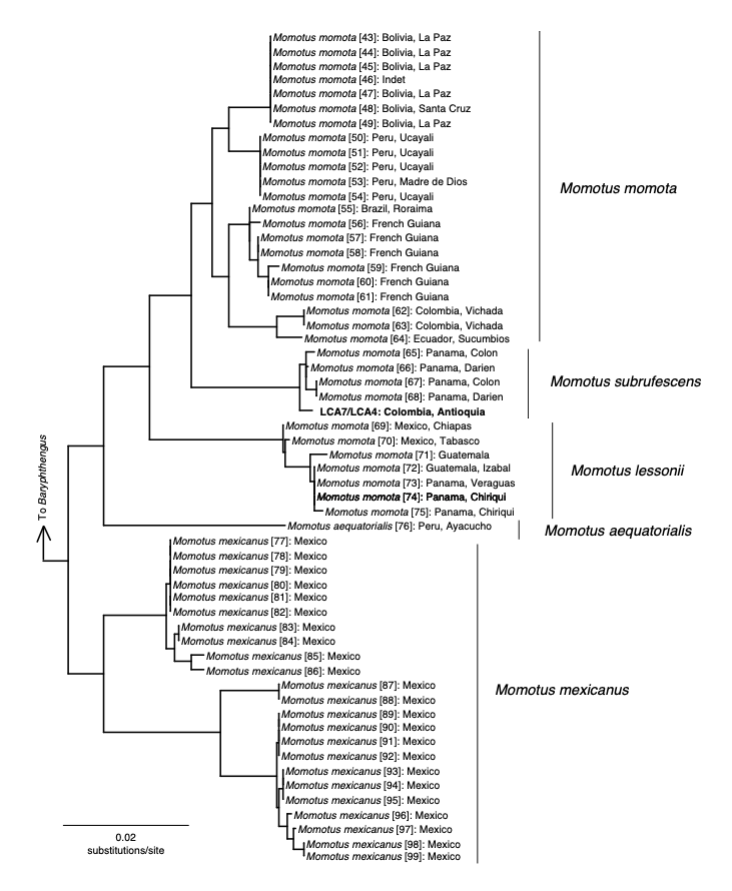
Kimura-2-parameter tree (obtained from the BOLD portal) of the "*Momotus momota* complex" showing the updated taxonomic arrangement of this clade. Numbers in brackets in the terminals correspond to BOLD numeric descriptors for each sample.

Most COI barcodes matched our initial phenotypic identification; however, for six (6) species, (10 individuals), we found differences between our field identification, the query hits from BOLD’s IDS and the NCBI BLAST search (Table 2). One bird species originally identified in the field as *Myiodynastes maculatus*, field ID LCA21) was positively identified as *Myiodynastes luteiventris*, (Fig. 2) by BOLD and NCBI analyses (but see Discussion). Another passerine species correctly identified in the field (field ID's LCA31, LCA36, LCA38) and by the NCBI BLAST as *Manacus manacus* (Fig. 3), was recovered as the Central American restricted species, *Manacus aurantiacus* by BOLD’s IDS. A third species was identified in the field (and NCBI BLAST search) as *Momotus subrufescens* (Fig. 4), but BOLD IDS recovered its former nominal assignation, *Momotus momota*, the name of a widely distributed form of motmot before it was split into five species-level taxa (Stiles 2009). Additionally, other three species (*Ramphocelus dimidiatus*, *Sporophila funerea* and *Xiphorhynchus susurrans*) were positively identified in the field and by BOLD, but exhibited erroneous identifications by the NCBI BLAST apparently because of the absence of COI sequences for either species in the latter portal. Finally, in six instances, DNA sequences helped to confirm the identification of *Automolus ochrolaemus*, *Chaetura brachyura* and *Tolmomyias sulphurescens*, which are all species difficult to identify in the field, even in hand, particularly the swifts.

## Discussion

Our assessment of species identification, using the COI barcodes, shows a strong correspondence (90%) with field identification, based on research expertise and photo ID (Table 2, Suppl. material 1). However, DNA barcodes were able to help with the identification of challenging species that can be problematic even for trained neotropical ornithologists. This was the case of field ID LCA21, identified initially as *Myiodynastes maculatus* (Hilty et al. 1986, Donegan and McMullan 2014), but for which both NCBI BLAST and the BOLD identification tool recovered it as *M. luteiventris* (Fig. 2). According to traditional and recent literature, some relevant diagnostic characters to identify *M. maculatus* include: insinuation or presence of rufous colouration on the margins of the primary feathers and coverts, a broadly pink lower-mandibular base (dark only the distal half), rufous tail with dark central stripe, narrow dusky malar stripe that does not meet under the bill (usually paler than that of *M. luteiventris*) and rufous or buffy supercilium (Shah 2020, Lowther and Stotz 2020, Donegan and McMullan 2014, Hilty et al. 1986, Ayerbe 2018). Although phenotypic characteristics of our specimen match those of *M. maculatus* (see Fig. 2), its COI sequence was grouped within the unique BIN containing *M. luteiventris* sequences (see Table 2). Moreover, its nearest neighbour is a *M. maculatus* BIN, with a strikingly large COI distance of 7.22%, reducing the possibility of a misidentification problem by BOLD's database. Considering this contradictory evidence (i.e. the phenotypic resemblance of our specimen with *M. maculatus* and the strong mitochondrial association with *M. luteiventris*), we cannot rule out the possibility of an introgression event, a phenomenon that has been documented amongst closely-related species with sympatric distributions in the family Tyrannidae (Ottenburghs et al. 2017, Rheindt et al. 2009, Rheindt et al. 2013, Winger 2017). Although evaluating a possible introgression scenario is outside of the scope of this study, it is important to highlight that barcoding studies can give us clues to understand these events.

Similarly, barcodes might help to identify the breeding areas or population origin for species exhibiting migratory divide or genetic structure, as happened with passing through northern South America species, *Catharus minimus* and *Catharus ustulatus* (Topp et al. 2013, Pulgarín-R et al. 2018). Additionally, barcodes can be of great help in resident species with little phenotypic variation, such as the swifts in the genus *Chaetura*, which are hard to capture in mist-nets and hard to identify in the field.

We also found some discrepancies between IDs recovered by the NCBI BLAST tool, those recovered by BOLD and our initial identifications made in the field. For example, three specimens identified in the field (Fig. 3) and by the NCBI BLAST tool as *Manacus manacus* were recovered by BOLD as *M. aurantiacus*. The BIN containing our sequences (Table 2) groups several phenotypes that, in the past, have been treated as the same species (e.g. Snow 1975) and also as a superspecies with up to four species (*M. aurantiacus*, *M. candei*, *M. manacus* and *M. vitellinus*) (Snow et al. 2004). Taxonomy within this group is not fully resolved so far that *M. aurantiacus* has been considered a subspecies of *M. vitellinus* (Snow 1975), an independent allopatrically-distributed species of the genus (Brumfield and Braun 2001, Brumfield et al. 2001,Brumfield et al. 2008) and even as a paraphyletic clade, based on mtDNA (Brumfield and Braun 2001). Moreover, it has been found that species of *Manacus* can hybridise in areas of sympatric distribution with other species of the genus and even the family (Brumfield and Braun 2001, Brumfield et al. 2001, Höglund and Shorey 2004). All the above-mentioned scenarios indicate that, although the phenotype of all our sequences corresponds to what is known as *M. manacus* (Fig. 3), in the absence of a clear phylogenetic arrangement and poor knowledge on the species limits within the genus *Manacus*, the DNA barcode, by itself, is not able to reconcile the morphological and molecular information and is only the reflection of a poorly understood taxonomy.

Another result that showed some inconsistencies was the identification of LCA4 and LCA7 sequences, which were recovered by BOLD as *Momotus momota* (Fig. 4). This used to be a widely-distributed species in Central and South America, until it was split into five species-level taxa (*M. aequatorialis*, *M. bahamensis*, *M. lessonii*, *M. momota* and *M. subrufescens*), using a combined analysis of plumage, biometrics and voice (Stiles 2009). Currently, *M. momota* is considered a cis-Andean distributed species from eastern Colombia to southern Venezuela, Guianas, north-western Argentina and most of Brazil (Stiles 2009). Particularly, the specific epithet, associated with the populations and phenotype obtained in this study, corresponds to *M. subrufescens*; however, despite the presence of eight different BINs that span much of the distribution of all the mentioned species within the genus, the taxonomy within the BOLD portal has yet to be updated and, consequently, our sequence is part of a BIN based on a haplotype with geographical proximity that bears the outdated *M. momota* taxon name.

A final group of inconsistencies between identification methods corresponds to three (3) species for which no COI sequence data are available at the NCBI portal and, consequently, their closest matching sequences are inconsistent with their correct field- and BOLD-based identifications. In the case of the genera *Ramphocelus* and *Xiphorhynchus*, the BLAST search tool identified our samples as the cis-Andean distributed congeneric species (*R. carbo* and *X. guttatus*) and not as the correct trans-Andean species, *R. dimidiatus* and *X. sussurrans* (Ayerbe 2018). For the genus *Sporophila*, although *S. angolensis* and *S. funerea* can show sympatric distributions (Ayerbe 2018), the morphology exhibited by their males is strikingly different and leaves no room for discussion on their morphological identification.

Even though we found some discrepancies between our identification methods compared to BOLD’s IDS, a close inspection to the K2P trees from BOLD (Fig. 4) showed that individuals across all sampled species are closely related to other individuals from nearby populations/areas. This is an important fact because, even in the presence of outdated or incorrect assignment of names to a barcode sequence (and, consequently, to its corresponding BIN), the K2P topologies are able to group individuals that, based even on geography itself, can putatively represent the current taxonomical treatment of the species (as is the case with the *Momotus subrufescens* in Fig. 4).

## Conclusions

Despite our reduced sampling, this study represents the first attempt to document COI barcodes for birds (from blood samples) in this part of Colombia, which fills a considerable gap in sampling in north-western South America. Particularly, a call for broader sampling for barcodes might provide hints on cryptic species across barriers (Barreira et al. 2016) or might facilitate the identification of highly-traded species in Colombia, such as parrots (Mendoza et al. 2016, Restrepo-R and Pulgarin-R 2017).

## Supplementary Material

0F790DA0-E3FA-586E-BA8F-C20218F6A7E2 10.3897/BDJ.9.e64842.suppl1 Supplementary material 1 Suplementary Information Data type DNA sequences, data Tables. File: oo_511293.docx https://binary.pensoft.net/file/511293 Pulgarin et al.

## Figures and Tables

**Table 1.** Primers used for the amplification of COI sequences obtained in this study.

**Name**	**Sequence + M13**	**Ratio**	**Source**
LepF1_t1-M13FWD	GTAAAACGACGGCCAGTATTCAACCAATCATAAAGATATTGG	1	Ivanova et al. 2007
VF1_t1-M13FWD	GTAAAACGACGGCCAGTTTCTCAACCAACCACAAAGACATTGG	1	Ivanova et al. 2007
VF1d_t1-M13FWD	GTAAAACGACGGCCAGTTTCTCAACCAACCACAARGAYATYGG	1	Ivanova et al. 2007
VF1i_t1-M13FWD	GTAAAACGACGGCCAGTTTCTCAACCAACCAIAAIGAIATIGG	3	Ivanova et al. 2007
LepRI_t1-M13REV	CAGGAAACAGCTATGACCTAAACTTCTGGATGTCCAAAAAATCA	1	Ivanova et al. 2007
VR1d_t1-M13REV	CAGGAAACAGCTATGACCTAGACTTCTGGGTGGCCRAARAAYCA	1	Ivanova et al. 2007
VR1_t1-M13REV	CAGGAAACAGCTATGACCTAGACTTCTGGGTGGCCAAAGAATCA	1	Ivanova et al. 2007
VR1i_t1-M13REV	CAGGAAACAGCTATGACCTAGACTTCTGGGTGICCIAAIAAICA	3	Ivanova et al. 2007
M13REV	CAGGAAACAGCTATGACC	NA	Beckman Coulter, Inc 2020
M13FWD	GTAAAACGACGGCCAGT	NA	Beckman Coulter, Inc 2020

**Table 2.** Individuals sampled and barcoded in this study. Individuals with * represent boreal migrants. Bolded taxa represent inconsistencies between our identification methods (see text).

**Code**	**Field ID**	**BOLD ID**	**BOLD hit^1^ (%)**	**NCBI ID**	**NCBI hit^1^(%)**	**Consensus sp BOLD ID**	**Seq length (bp)**	**BIN**
LCA35	*Automolus ochrolaemus*	*A. ochrolaemus*	100	*A. ochrolaemus*	99.10	*A. ochrolaemus*	671	BOLD:ADM4531
LCA9	*Cantorchilus leucotis*	*C. leucotis*	100	*C. leucotis*	95.55	*C. leucotis*	690	BOLD:ABX4224
LCA12	*Catharus minimus*	*C. minimus*	100	*C. minimus*	100	C. minimus*	657	BOLD:AAA9441
LCA30	*Catharus minimus*	*C. minimus*	100	C. minimus*	100	C. minimus*	660	BOLD:AAA9441
LCA3	*Catharus ustulatus*	C. ustulatus*	100	C. ustulatus*	100	C. ustulatus*	702	BOLD:AAA9440
LCA26	*Chaetura* sp	*C. brachyura*	100	*C. brachyura*	100	*C. brachyura*	644	BOLD:AAK0488
LCA27	*Chaetura* sp	*C. brachyura*	100	*C. brachyura*	100	*C. brachyura*	642	BOLD:AAK0488
LCA28	*Chaetura* sp	*C. brachyura*	100	*C. brachyura*	100	*C. brachyura*	652	BOLD:AAK0488
LCA24	*Coereba flaveola*	*C. flaveola*	100	*C. flaveola*	100	*C. flaveola*	651	BOLD:AAA4006
LCA33	*Dendrocincla fuliginosa*	*D. fuliginosa*	99.85	*D. fuliginosa*	99.15	*D. fuliginosa*	673	BOLD:ABZ6107
LCA20	*Elaenia flavogaster*	*E. flavogaster*	99.85	*E. flavogaster*	98.93	*E. flavogaster*	681	BOLD:AAB3859
LCA6	*Elaenia flavogaster*	*E. flavogaster*	100	*E. flavogaster*	99.39	*E. flavogaster*	696	BOLD:AAB3859
LCA18	*Galbula ruficauda*	*G. ruficauda*	100	*G. ruficauda*	97.55	*G. ruficauda*	675	BOLD:ABX4491
LCA31	***Manacus manacus***	***M. aurantiacus***	100	***M. manacus***	100	***M. aurantiacus***	667	BOLD:AAB9291
LCA36	***Manacus manacus***	***M. aurantiacus***	100	***M. manacus***	100	***M. aurantiacus***	663	BOLD:AAB9291
LCA38	***Manacus manacus***	***M. aurantiacus***	100	***M. manacus***	100	***M. aurantiacus***	667	BOLD:AAB9291
LCA4	***Momotus subrufescens***	***M. momota***	100	***M. momota***	96.92	***M. momota***	681	BOLD:ABX4186
LCA7	***Momotus subrufescens***	***M. momota***	100	***M. momota***	97.41	***M. momota***	657	BOLD:ABX4186
LCA21	***Myiodinastes maculatus***	***M. luteiventris***	100	M. luteiventris*	100	M. luteiventris*	651	BOLD:AAF5348
LCA22	*Myiozetetes cayanensis*	*M. cayanensis*	99.85	*M. cayanensis*	98.77	*M. cayanensis*	660	BOLD:AAE6211
LCA13	*Parkesia noveboracensis*	*P. noveboracensis*	100	*P. noveboracensis*	99.85	P. noveboracensis*	658	BOLD:AAB0401
LCA15	***Ramphocelus dimidiatus***	***R. dimidiatus***	100	***R. carbo***	99.39	***R. dimidiatus***	681	BOLD:AAD5047
LCA40	***Ramphocelus dimidiatus***	***R. dimidiatus***	100	***R. carbo***	99.23	***R. dimidiatus***	654	BOLD:AAD5047
LCA1	***Sporophila funerea***	***S. funerea***	100	***S. angloennsis***	98.92	***S. funerea***	687	BOLD:AAE5360
LCA19	*Tolmomyias sulphurescens*	*T. sulphurescens*	99.85	*T. sulphurescens*	97.89	*T. sulphurescens*	666	BOLD:ACI3658
LCA8	***Xiphorhynchus susurrans***	***X. susurrans***	99.54	***X. guttatus***	98.15	***X. susurrans***	670	BOLD:ACF1637
