# Supplementary material for: DNA barcodes of birds from northern Colombia
Source: Biodivers Data J. 2021 May 21;9:e64842. doi: 10.3897/BDJ.9.e64842 (PMC8163714; doi:10.3897/BDJ.9.e64842)
Supplement: Supplementary material 1 — Suplementary Information [file bdj-09-e64842-s001.docx]

Supporting information S1

Table S1: Query hits from the BOLD Species Level Barcode Records identification tool (http://www.boldsystems.org/index.php/IDS_OpenIdEngine)

| **Query ID** | **Best ID** | **Search DB** | **Top %** | **Low %** |
| --- | --- | --- | --- | --- |
| LCA40_F_R_Ramphocellus_dimidiatus | *Ramphocelus dimidiatus* | COI SPECIES DATABASE | 99.85 | 91.38 |
| LCA38_F_R_Manacus_manacus | *Manacus aurantiacus* | COI SPECIES DATABASE | 99.49 | 91.55 |
| LCA36_F_R_Manacus_manacus | *Manacus aurantiacus* | COI SPECIES DATABASE | 100 | 91.67 |
| LCA35_F_R_Automolus_ochrolaemus | *Automolus ochrolaemus* | COI SPECIES DATABASE | 100 | 89.33 |
| LCA33_F_R_Dendrocincla_fuliginosa | *Dendrocincla fuliginosa* | COI SPECIES DATABASE | 99.85 | 93.97 |
| LCA31_F_R_Manacus_manacus | *Manacus aurantiacus* | COI SPECIES DATABASE | 100 | 91.55 |
| LCA30_F_R_Catharus_minimus | *Catharus minimus* | COI SPECIES DATABASE | 100 | 93.06 |
| LCA28_F_R_Chaetura_brachyura | *Chaetura brachyura* | COI SPECIES DATABASE | 100 | 89.66 |
| LCA27_F_R_Chaetura_brachyura | *Chaetura brachyura* | COI SPECIES DATABASE | 100 | 89.55 |
| LCA26_F_R_Chaetura_brachyura | *Chaetura brachyura* | COI SPECIES DATABASE | 100 | 89.51 |
| LCA24_F_R_Coereba_flaveola | *Coereba flaveola* | COI SPECIES DATABASE | 100 | 97.36 |
| LCA22_F_R_Myiozetetes_cayanensis | *Myiozetetes cayanensis* | COI SPECIES DATABASE | 100 | 89.40 |
| LCA21_F_R_Myiodynastes_maculatus | *Myiodynastes luteiventris* | COI SPECIES DATABASE | 100 | 89.07 |
| LCA20_F_R_Elaenia_flavogaster | *Elaenia flavogaster* | COI SPECIES DATABASE | 99.85 | 90.28 |
| LCA19_F_R_Tolmomyias_sulphurescens | *Tolmomyias sulphurescens* | COI SPECIES DATABASE | 99.85 | 86.54 |
| LCA18_F_R Galbula_ruficauda | *Galbula ruficauda* | COI SPECIES DATABASE | 100 | 85.50 |
| LCA15_F_R_Ramphocelus_dimidiatus | *Ramphocelus dimidiatus* | COI SPECIES DATABASE | 100 | 91.19 |
| LCA13_F_R_ Parkesia_novevoracensis | *Parkesia noveboracensis* | COI SPECIES DATABASE | 100 | 93.35 |
| LCA12_F_R_Catharus_minimus | *Catharus minimus* | COI SPECIES DATABASE | 100 | 93.95 |
| LCA9_F_R_Cantorchilus_leucotis | *Cantorchilus leucotis* | COI SPECIES DATABASE | 98.92 | 91.25 |
| LCA8_F_R_Xiphorhynchus_susurrans | *Xiphorhynchus susurrans* | COI SPECIES DATABASE | 99.53 | 91.44 |
| LCA7_F_R_Momotus_subrufescens | *Momotus momota* | COI SPECIES DATABASE | 99.69 | 87.00 |
| LCA6_F_R_Elaenia_flavogaster | *Elaenia flavogaster* | COI SPECIES DATABASE | 100 | 90.12 |
| LCA4_F_R_Momotus_subrufescens | *Momotus momota* | COI SPECIES DATABASE | 99.69 | 87.00 |
| LCA3_F_R_Catharus_ustulatus | *Catharus ustulatus* | COI SPECIES DATABASE | 100 | 92.52 |
| LCA1_F_R_Oryzoborus_funereus | *Oryzoborus funereus* | COI SPECIES DATABASE | 100 | 92.15 |

**Table S2.** Individuals sampled and barcoded in this study. Individuals with * represent boreal migrants. Bolded taxa represent inconsistencies between our identification methods (see text).

| **Code** | **Date** | **Field ID** | **Age** | **Sex** | **weight (g)** | **wing (mm)** | **tarsus (right)** | **tail (mm)** | **Seq length (bp)** | **BIN** |
| --- | --- | --- | --- | --- | --- | --- | --- | --- | --- | --- |
| LCA35 | 29-Oct-17 | *Automolus ochrolaemus* | adult | na | 34,5 | 90 | 21,9 | 76 | 671 | [BOLD:ADM4531](https://www.boldsystems.org/index.php/Public_BarcodeCluster?clusteruri=BOLD:ADM4531) |
| LCA9 | 27-Oct-17 | *Cantorchilus leucotis* | adult | na | 19 | 67 | 23,8 | 43 | 690 | [BOLD:ABX4224](https://www.boldsystems.org/index.php/Public_BarcodeCluster?clusteruri=BOLD:ABX4224) |
| LCA12 | 27-Oct-17 | *Catharus minimus* | juvenil | na | 32 | 110 | 30,5 | 77 | 657 | [BOLD:AAA9441](https://www.boldsystems.org/index.php/Public_BarcodeCluster?clusteruri=BOLD:AAA9441) |
| LCA30 | 28-Oct-17 | *Catharus minimus* | adult | na | 31 | 110 | 30 | 73 | 660 | [BOLD:AAA9441](https://www.boldsystems.org/index.php/Public_BarcodeCluster?clusteruri=BOLD:AAA9441) |
| LCA3 | 26-Oct-17 | *Catharus ustulatus* | juvenil | na | 34 | 101 | 29,7 | 69 | 702 | [BOLD:AAA9440](https://www.boldsystems.org/index.php/Public_BarcodeCluster?clusteruri=BOLD:AAA9440) |
| LCA26 | 27-Oct-17 | *Chaetura sp* | adult | na | 20,5 | 121 | 12,2 | 32 | 644 | [BOLD:AAK0488](https://www.boldsystems.org/index.php/Public_BarcodeCluster?clusteruri=BOLD:AAK0488) |
| LCA27 | 27-Oct-17 | *Chaetura sp* | adult | na | 19,5 | 125 | 12,5 | 31 | 642 | [BOLD:AAK0488](https://www.boldsystems.org/index.php/Public_BarcodeCluster?clusteruri=BOLD:AAK0488) |
| LCA28 | 27-Oct-17 | *Chaetura sp* | adult | na | 23 | 112 | 12,5 | 30 | 652 | [BOLD:AAK0488](https://www.boldsystems.org/index.php/Public_BarcodeCluster?clusteruri=BOLD:AAK0488) |
| LCA24 | 27-Oct-17 | *Coereba flaveola* | adult | na | na | 56 | 17,5 | 36 | 651 | [BOLD:AAA4006](https://www.boldsystems.org/index.php/Public_BarcodeCluster?clusteruri=BOLD:AAA4006) |
| LCA33 | 28-Oct-17 | *Dendrocincla fuliginosa* | juvenil | na | 37 | 105 | 25,8 | 88 | 673 | [BOLD:ABZ6107](https://www.boldsystems.org/index.php/Public_BarcodeCluster?clusteruri=BOLD:ABZ6107) |
| LCA20 | 27-Oct-17 | *Elaenia flavogaster* | adult | na | 20,5 | 75 | 19,3 | 79 | 681 | [BOLD:AAB3859](https://www.boldsystems.org/index.php/Public_BarcodeCluster?clusteruri=BOLD:AAB3859) |
| LCA6 | 26-Oct-17 | *Elaenia flavogaster* | adult | na | 24 | 80 | 21,7 | 76 | 696 | [BOLD:AAB3859](https://www.boldsystems.org/index.php/Public_BarcodeCluster?clusteruri=BOLD:AAB3859) |
| LCA18 | 27-Oct-17 | *Galbula ruficauda* | adult | na | na | 82 | 14,1 | 108 | 675 | [BOLD:ABX4491](https://www.boldsystems.org/index.php/Public_BarcodeCluster?clusteruri=BOLD:ABX4491) |
| LCA31 | 28-Oct-17 | *Manacus manacus* | adult | male | na | na | na | na | 667 | [BOLD:AAB9291](https://www.boldsystems.org/index.php/Public_BarcodeCluster?clusteruri=BOLD:AAB9291) |
| LCA36 | 29-Oct-17 | *Manacus manacus* | adult | female | 15 | 55 | 19,8 | 31 | 663 | [BOLD:AAB9291](https://www.boldsystems.org/index.php/Public_BarcodeCluster?clusteruri=BOLD:AAB9291) |
| LCA38 | 29-Oct-17 | *Manacus manacus* | adult | male | 19 | 55 | 22,3 | 30 | 667 | [BOLD:AAB9291](https://www.boldsystems.org/index.php/Public_BarcodeCluster?clusteruri=BOLD:AAB9291) |
| LCA4 | 26-Oct-17 | *Momotus subrufescens* | adult | na | 95 | 129 | 31,6 | 250 | 681 | [BOLD:ABX4186](https://www.boldsystems.org/index.php/Public_BarcodeCluster?clusteruri=BOLD:ABX4186) |
| LCA7 | 26-Oct-17 | *Momotus subrufescens* | adult | na | 120 | 132 | na | 240 | 657 | [BOLD:ABX4186](https://www.boldsystems.org/index.php/Public_BarcodeCluster?clusteruri=BOLD:ABX4186) |
| LCA21 | 27-Oct-17 | *Myiodinastes maculatus* | juvenil | na | 40,5 | 110 | 19,1 | 82 | 651 | [BOLD:AAF5348](https://www.boldsystems.org/index.php/Public_BarcodeCluster?clusteruri=BOLD:AAF5348) |
| LCA22 | 27-Oct-17 | *Myiozetetes cayanensis* | adult | na | 26 | 91 | 18,5 | 75 | 660 | [BOLD:AAE6211](https://www.boldsystems.org/index.php/Public_BarcodeCluster?clusteruri=BOLD:AAE6211) |
| LCA13 | 27-Oct-17 | *Parkesia novevoracensis* | juvenil | na | 15,4 | 79 | 22,1 | 55 | 658 | [BOLD:AAB0401](https://www.boldsystems.org/index.php/Public_BarcodeCluster?clusteruri=BOLD:AAB0401) |
| LCA15 | 27-Oct-17 | *Ramphocellus dimidiatus* | juvenil | female | 23,3 | 74 | 19,5 | 67 | 681 | [BOLD:AAD5047](https://www.boldsystems.org/index.php/Public_BarcodeCluster?clusteruri=BOLD:AAD5047) |
| LCA40 | 29-Oct-17 | *Ramphocellus dimidiatus* | adult | na | 24 | 75 | 19,7 | 67 | 654 | [BOLD:AAD5047](https://www.boldsystems.org/index.php/Public_BarcodeCluster?clusteruri=BOLD:AAD5047) |
| LCA1 | 26-Oct-17 | *Sporophila funerea* | juvenil | male | 12,5 | 56 | 16 | - | 687 | [BOLD:AAE5360](https://www.boldsystems.org/index.php/Public_BarcodeCluster?clusteruri=BOLD:AAE5360) |
| LCA19 | 27-Oct-17 | *Tolmomyias sulphurescens* | adult | na | 16 | 66 | 17,7 | 58 | 666 | [BOLD:ACI3658](https://www.boldsystems.org/index.php/Public_BarcodeCluster?clusteruri=BOLD:ACI3658) |
| LCA8 | 27-Oct-17 | *Xiphorhynchus susurrans* | adult | na | 51 | 108 | 25,2 | 94 | 670 | [BOLD:ACF1637](https://www.boldsystems.org/index.php/Public_BarcodeCluster?clusteruri=BOLD:ACF1637) |

Supporting information S2 - Sequence information on fasta format for all the 26 individuals sampled and barcoded in La Candelaria.

>LCA40_F_R_Ramphocellus_dimidiatus

CTGTACCTAATTTTCGGTGCATGAGCCGGAATAGTGGGTACTGCCCTAAGCCTCCTCATTCGAGCAGAACTAGGCCAACCTGGAGCTCTCCTGGGAGACGACCAAGTCTACAACGTGGTCGTCACAGCCCATGCCTTCGTAATAATCTTCTTCATAGTTATGCCAATTATGATCGGGGGATTCGGGAACTGACTAGTCCCATTAATAATTGGAGCCCCAGACATAGCATTCCCACGGATAAACAACATAAGCTTTTGGCTACTTCCCCCATCTTTCCTTCTCCTCCTAGCATCTTCCACAGTCGAAGCAGGGGTCGGTACAGGTTGAACAGTATATCCCCCACTAGCTGGCAACCTAGCCCACGCCGGAGCCTCAGTCGACTTAGCAATCTTCTCCCTACATCTAGCTGGCATCTCTTCAATCCTTGGAGCAATCAACTTCATCACAACAGCAATCAACATGAAACCTCCCGCTCTGTCACAATACCAAACCCCCCTATTCGTATGATCAGTATTAATCACTGCAGTCTTACTGCTCCTATCTCTTCCAGTCCTTGCTGCAGGAATTACAATGCTCCTGACGGACCGCAACCTTAACACTACATTCTTCGACCCCGCTGGAGGAGGAGACCCCGTACTATACCAACACCTTTTC

>LCA38_F_R_Manacus_manacus

GGAGATGGGCACACTTTACCTTATTTTTGGCGCATGAGCTGGTATAATCGGCACAGCCCTTAGTCTCCTCATTCGAGCAGAACTAGGACAGCCAGGCACCCTTTTAGGCGATGACCAAATTTACAATGTAATCGTCACCGCACATGCCTTCGTTATAATTTTCTTTATAGTAATGCCTATTATAATCGGAGGGTTTGGTAACTGATTAGTACCATTAATAATTGGTGCCCCCGACATAGCATTTCCACGAATAAATAACATGAGCTTCTGACTACTTCCTCCATCCTTCCTACTTCTTCTAGCATCTTCTACAGTAGAAGCCGGGGCCGGAACAGGATGAACAGTATACCCACCCCTAGCCGGAAATCTAGCCCACGCCGGTGCATCAGTAGACCTTGCCATCTTCTCCCTTCACCTGGCAGGTGTCTCATCCATCCTTGGGGCAATCAACTTTATCACCACAGCAATCAATATAAAACCACCTGCTCTCTCACAATACCAAACCCCTCTATTCGTTTGATCCGTCCTAATTACTGCAGTTCTACTTCTCCTCTCCCTACCAGTCCTCGCTGCTGGCATTACAATGCTTCTAACAGATCGAAACCTAAACACTACATTCTTTGACCCCGCAGGAGGCGGAGACCCAATTTTATATCAACACTTATTC

>LCA36_F_R_Manacus_manacus

ATGGGCACACTTTACCTTATTTTTGGCGCATGAGCTGGTATAATCGGCACAGCCCTTAGTCTCCTCATTCGAGCAGAACTAGGACAGCCAGGCACCCTTTTAGGCGATGACCAAATTTACAATGTAATCGTCACCGCACATGCCTTCGTTATAATTTTCTTTATAGTAATGCCTATTATAATCGGAGGGTTTGGTAACTGATTAGTACCATTAATAATTGGTGCCCCCGACATAGCATTTCCACGAATAAATAACATGAGCTTCTGACTACTTCCTCCATCCTTCCTACTTCTTCTAGCATCTTCTACAGTAGAAGCCGGGGCCGGAACAGGATGAACAGTATACCCACCCCTAGCCGGAAATCTAGCCCACGCCGGTGCATCAGTAGACCTTGCCATCTTCTCCCTTCACCTGGCAGGTGTCTCATCCATCCTTGGGGCAATCAACTTTATCACCACAGCAATCAATATAAAACCACCTGCTCTCTCACAATACCAAACCCCTCTATTCGTTTGATCCGTCCTAATTACTGCAGTTCTACTTCTCCTCTCCCTACCAGTCCTCGCTGCTGGCATTACAATGCTTCTAACAGATCGAAACCTAAACACTACATTCTTTGACCCCGCAGGAGGCGGAGACCCAATTTTATATCAACACTTATTC

>LCA35_F_R_Automolus_ochrolaemus

AGAAGGAGATGGGCACTCTCTACCTGATTTTTGGGGCATGAGCCGGTATAATTGGAACCTCCCTTAGTCTCCTAATTCGAGCTGAACTTGGACAACCAGGCACTCTCCTGGGTGATGACCAAATTTATAATGTTATTGTCACCGCCCATGCCTTCGTAATAATCTTTTTCATAGTTATACCCATTATAATCGGCGGCTTCGGTAACTGATTAGTCCCACTAATAATTGGCGCCCCCGACATAGCTTTCCCCCGAATAAATAACATAAGCTTTTGACTCCTACCCCCATCCTTCCTGCTCCTACTAGCTTCCTCAACAGTAGAAGCCGGAGCAGGAACAGGATGGACAGTCTACCCACCATTAGCAGGCAACCTGGCTCACGCTGGAGCCTCAGTAGACCTAGCTATCTTCTCCCTTCATCTAGCAGGTGTATCCTCTATTCTAGGAGCTATCAACTTTATTACAACCGCAATCAACATAAAACCCCCCGCTCTCTCACAATACCAAACCCCACTATTCGTTTGATCCGTCCTCATTACTGCCGTTCTACTCCTACTCTCCCTTCCTGTCCTAGCTGCTGGAATCACAATATTACTCACAGACCGCAATCTTAACACCACATTCTTTGACCCTGCCGGAGGAGGAGACCCTGTACTATACCAACACTTATTC

>LCA33_F_R_Dendrocincla_fuliginosa

CCAGAAGGAGATGGGCACCCTATATCTAATCTTCGGGGCATGAGCCGGAATAATTGGAACCGCTCTAAGCCTCCTAATCCGAGCCGAACTTGGACAACCAGGTACCCTCCTAGGTGATGACCAAATCTATAACGTCATCGTAACCGCCCATGCTTTCGTAATAATTTTCTTTATAGTTATACCTATCATAATTGGAGGATTTGGCAACTGACTAGTTCCACTAATAATTGGTGCTCCCGACATAGCATTCCCACGAATAAATAACATAAGCTTCTGACTCCTACCCCCATCCTTTCTTCTACTTCTAGCTTCCTCTACAGTAGAAGCAGGTGCAGGAACAGGATGAACAGTATACCCGCCCCTAGCAGGTAATCTAGCCCACGCAGGAGCCTCAGTAGATTTAGCTATCTTTTCTCTTCACTTAGCTGGTGTATCCTCTATTCTAGGGGCAATCAATTTTATCACAACTGCAATCAACATAAAACCACCAGCCCTCTCGCAATACCAAACCCCCCTATTTGTTTGATCCGTCCTTATCACCGCTGTCTTACTTCTTCTATCTCTACCTGTTCTAGCAGCCGGTATCACAATACTCTTAACAGACCGTAACCTAAATACTACATTCTTCGACCCAGCTGGAGGCGGAGACCCTATCCTATACCAACATCTATTC

>LCA31_F_R_Manacus_manacus

GGAGATGGGCACACTTTACCTTATTTTTGGCGCATGAGCTGGTATAATCGGCACAGCCCTTAGTCTCCTCATTCGAGCAGAACTAGGACAGCCAGGCACCCTTTTAGGCGATGACCAAATTTACAATGTAATCGTCACCGCACATGCCTTCGTTATAATTTTCTTTATAGTAATGCCTATTATAATCGGAGGGTTTGGTAACTGATTAGTACCATTAATAATTGGTGCCCCCGACATAGCATTTCCACGAATAAATAACATGAGCTTCTGACTACTTCCTCCATCCTTCCTACTTCTTCTAGCATCTTCTACAGTAGAAGCCGGGGCCGGAACAGGATGAACAGTATACCCACCCCTAGCCGGAAATCTAGCCCACGCCGGTGCATCAGTAGACCTTGCCATCTTCTCCCTTCACCTGGCAGGTGTCTCATCCATCCTTGGGGCAATCAACTTTATCACCACAGCAATCAATATAAAACCACCTGCTCTCTCACAATACCAAACCCCTCTATTCGTTTGATCCGTCCTAATTACTGCAGTTCTACTTCTCCTCTCCCTACCAGTCCTCGCTGCTGGCATTACAATGCTTCTAACAGATCGAAACCTAAACACTACATTCTTTGACCCCGCAGGAGGCGGAGACCCAATTTTATATCAACACTTATTC

>LCA30_F_R_Catharus_minimus

GGTACCCTCTACCTAATTTTCGGCGCGTGAGCCGGAATAGTGGGCACCGCCCTAAGTCTTCTCATCCGAGCAGAACTGGGTCAACCAGGCGCACTACTAGGCGATGACCAGATCTACAACGTAGTTGTCACTGCTCATGCCTTCGTAATAATTTTCTTTATAGTCATGCCAATCATGATTGGAGGGTTCGGAAACTGACTAGTCCCCTTAATAATCGGAGCCCCAGACATAGCATTCCCTCGAATAAACAACATAAGCTTCTGACTTCTTCCACCATCATTCCTCCTCCTCTTAGCCTCCTCCACAGTAGAAGCAGGAGCAGGAACAGGATGAACCGTCTATCCCCCCCTTGCCGGCAACCTAGCACACGCAGGAGCCTCAGTAGACCTGGCTATCTTCTCCCTCCACTTAGCAGGAATCTCCTCAATCCTAGGGGCCATCAACTTCATTACCACAGCTATCAACATAAAACCTCCCGCCCTATCACAATACCAAACCCCCCTATTCGTATGATCAGTACTAATCACTGCAGTCTTACTCCTCCTCTCCCTTCCCGTCCTTGCTGCTGGCATCACCATACTCCTCACCGACCGTAACCTAAACACCACCTTCTTCGACCCAGCAGGAGGAGGAGACCCAGTACTTTACCAGCATCTATTC

>LCA28_F_R_Chaetura_brachyura

GTACTTAATCTTTGGAGCATGAGCTGGCATAGTAGGTACCGCCCTCAGCCTACTCATCCGAGCAGAACTTGGACAACCAGGGACCCTCCTGGGAGACGATCAAATTTACAACGTAATCGTCACTGCTCACGCCTTCGTAATAATCTTCTTCATAGTTATACCAATTATGATTGGAGGATTTGGAAACTGACTAGTCCCACTTATAATTGGAGCACCTGACATAGCCTTCCCACGAATAAATAATATAAGCTTCTGACTCCTTCCCCCATCATTCCTTCTCCTACTAGCCTCCTCAACAGTTGAAGCAGGAGCAGGAACAGGCTGAACCGTATACCCCCCACTAGCCGGCAATCTAGCCCATGCAGGAGCATCAGTAGACCTCGCCATCTTCTCCCTCCACCTAGCAGGTGTCTCCTCCATCCTAGGTGCAATCAACTTCATCACAACTGCCATCAATATAAAACCACCCGCCCTTTCACAATACCAAACACCCCTATTCGTATGATCCGTCCTCATTACCGCCGTCCTACTACTCCTCTCCCTCCCTGTCCTCGCCGCAGGCATCACCATACTCTTAACCGACCGCAACCTAAACACCACATTCTTCGACCCAGCCGGAGGAGGTGACCCCATCCTCTACCAACACCTATTC

>LCA27_F_R_Chaetura_brachyura

TTTGGAGCATGAGCTGGCATAGTAGGTACCGCCCTCAGCCTACTCATCCGAGCAGAACTTGGACAACCAGGGACCCTCCTGGGAGACGATCAAATTTACAACGTAATCGTCACTGCTCACGCCTTCGTAATAATCTTCTTCATAGTTATACCAATTATGATTGGAGGATTTGGAAACTGACTAGTCCCACTTATAATTGGAGCACCTGACATAGCCTTCCCACGAATAAATAATATAAGCTTCTGACTCCTTCCCCCATCATTCCTTCTCCTACTAGCCTCCTCAACAGTTGAAGCAGGAGCAGGAACAGGCTGAACCGTATACCCCCCACTAGCCGGCAATCTAGCCCATGCAGGAGCATCAGTAGACCTCGCCATCTTCTCCCTCCACCTAGCAGGTGTCTCCTCCATCCTAGGTGCAATCAACTTCATCACAACTGCCATCAATATAAAACCACCCGCCCTTTCACAATACCAAACACCCCTATTCGTATGATCCGTCCTCATTACCGCCGTCCTACTACTCCTCTCCCTCCCTGTCCTCGCCGCAGGCATCACCATACTCTTAACCGACCGCAACCTAAACACCACATTCTTCGACCCAGCCGGAGGAGGTGACCCCATCCTCTACCAACACCTATTC

>LCA26_F_R_Chaetura_brachyura

TCTTTGGAGCATGAGCTGGCATAGTAGGTACCGCCCTCAGCCTACTCATCCGAGCAGAACTTGGACAACCAGGGACCCTCCTGGGAGACGATCAAATTTACAACGTAATCGTCACTGCTCACGCCTTCGTAATAATCTTCTTCATAGTTATACCAATTATGATTGGAGGATTTGGAAACTGACTAGTCCCACTTATAATTGGAGCACCTGACATAGCCTTCCCACGAATAAATAATATAAGCTTCTGACTCCTTCCCCCATCATTCCTTCTCCTACTAGCCTCCTCAACAGTTGAAGCAGGAGCAGGAACAGGCTGAACCGTATACCCCCCACTAGCCGGCAATCTAGCCCATGCAGGAGCATCAGTAGACCTCGCCATCTTCTCCCTCCACCTAGCAGGTGTCTCCTCCATCCTAGGTGCAATCAACTTCATCACAACTGCCATCAATATAAAACCACCCGCCCTTTCACAATACCAAACACCCCTATTCGTATGATCCGTCCTCATTACCGCCGTCCTACTACTCCTCTCCCTCCCTGTCCTCGCCGCAGGCATCACCATACTCTTAACCGACCGCAACCTAAACACCACATTCTTCGACCCAGCCGGAGGAGGTGACCCCATCCTCTACCAACACCTATTC

>LCA24_F_R_Coereba_flaveola

TACCTAATCTTCGGTGCATGAGCCGGAATAGTAGGCACAGCCCTAAGCCTCCTCATTCGATCAGAGCTAGGTCAACCCGGAGCCCTCCTAGGAGACGACCAAGTCTACAACGTAGTCGTCACAGCCCACGCCTTTGTAATAATCTTCTTCATAGTTATACCAATCATGATTGGAGGCTTTGGTAACTGACTAGTCCCCCTAATAATCGGAGCCCCAGACATAGCATTTCCACGAATAAACAACATAAGCTTCTGACTACTCCCCCCATCCTTTCTCCTCCTCCTAGCATCTTCCACAGTTGAAGCAGGCGTCGGTACCGGTTGAACAGTATATCCCCCATTAGCCGGCAACCTAGCCCATGCCGGAGCATCAGTAGACCTGGCAATCTTCTCCCTTCACCTGGCCGGAATTTCCTCAATCCTAGGGGCAATCAACTTTATCACAACTGCCGTCAATATGAAACCCCCTGCCCTCTCACAATACCAAACCCCCCTATTCGTCTGATCCGTCCTAATCACCGCAGTCCTGCTACTCCTATCCCTCCCAGTCCTAGCTGCTGGAATTACGATACTCCTTACAGACCGCAACCTCAATACTACATTCTTCGACCCTGCAGGAGGAGGAGACCCAGTCCTATATCAACACCTTTTC

>LCA22_F_R_Myiozetetes_cayanensis

GGTACCCTATACTTAATTTTCGGCGCCTGAGCCGGCATGATTGGTACTGCCCTAAGTCTCCTTATCCGAGCAGAACTTGGACAACCAGGAACCCTTCTAGGAGACGACCAAATCTATAATGTAATTGTTACCGCCCATGCTTTTGTAATAATCTTCTTTATAGTAATACCTATTATAATTGGAGGATTTGGCAACTGACTAGTCCCCCTAATAATTGGTGCCCCCGATATGGCATTCCCACGCATGAACAACATAAGTTTCTGACTTCTTCCCCCATCATTCCTTCTCCTCCTAGCCTCATCTACAATTGAAGCCGGGGTAGGAACTGGATGAACCGTATACCCACCATTAGCTGGCAATCTAGCACATGCTGGAGCTTCAGTAGACCTGGCCATCTTCTCACTTCACCTTGCAGGCATTTCCTCAATCCTAGGTGCCATCAACTTCATTACCACTGCAATTAACATGAAACCACCAGCCCTATCACAATATCAAACCCCCTTGTTCGTATGATCTGTCCTAATCACCGCAGTTCTTCTTCTCCTCTCTCTACCAGTTCTCGCTGCTGGTATTACCATACTCCTGACAGACCGCAATCTCAACACTACATTCTTTGACCCCGCAGGAGGTGGAGATCCAGTTCTATACCAACACCTCTTC

>LCA21_F_R_Myiodynastes_luteiventris

TATTTAATTTTTGGCGCCTGAGCCGGTATAATTGGTACCGCCTTAAGCCTTCTTATCCGAGCAGAACTAGGACAGCCAGGAACTCTCCTGGGAGATGACCAAATCTATAACGTAATCGTCACTGCTCATGCTTTCGTAATAATCTTTTTTATAGTTATACCTATCATAATCGGGGGATTCGGAAATTGACTAGTTCCCCTAATAATTGGCGCCCCAGACATAGCATTCCCACGAATGAACAATATAAGCTTTTGACTACTCCCCCCATCCTTCCTTCTCCTCCTAGCTTCATCCACAGTTGAAGCTGGAGTAGGAACCGGGTGAACTGTCTACCCACCATTAGCTGGTAACCTAGCACACGCTGGAGCTTCAGTAGACCTGGCCATCTTCTCCCTTCACCTCGCAGGCGTTTCTTCAATCTTAGGTGCTATCAATTTTATTACCACTGCAATTAACATAAAACCCCCAGCCCTAACACAATATCAAACCCCTCTATTCGTCTGATCAGTCTTAATCACTGCAGTTCTTCTCCTCCTCTCCCTCCCAGTCCTCGCTGCTGGCATCACTATATTATTAACAGATCGTAACCTTAATACCACATTCTTTGACCCAGCAGGAGGCGGAGACCCAGTCCTATATCAACACCTATTC

>LCA20_F_R_Elaenia_flavogaster

TCAACCAACCAGAAGGAGATGGGTACATTATACCTAATTTTCGGTGCCTGAGCCGGCATAATTGGCACTGCCCTAAGCCTTCTTATCCGAGCAGAACTTGGTCAACCCGGAACACTCCTAGGAGATGACCAGATCTATAACGTAATTGTTACCGCCCATGCCTTTGTAATAATCTTCTTCATAGTTATACCTATCATAATTGGAGGATTCGGCAACTGACTAGTCCCCCTGATAATTGGAGCCCCTGACATAGCATTCCCACGTATAAATAACATAAGTTTTTGACTCCTTCCCCCATCATTTCTTCTCCTCTTAGCCTCGTCCACAGTTGAAGCCGGGGTAGGAACGGGATGAACCGTTTACCCACCATTAGCCGGCAACCTAGCCCATGCAGGTGCTTCAGTAGACTTAGCTATCTTCTCCCTCCACCTTGCAGGAGTGTCTTCAATCCTAGGTGCTATTAATTTTATTACCACCGCAATCAATATGAAACCACCAGCCCTCTCACAATACCAAACACCTCTATTTGTTTGATCCGTCCTAATTACCGCAGTCCTTCTTCTCCTCTCTCTCCCTGTTCTCGCTGCTGGCATTACCATGCTTCTAACAGACCGAAACCTTAACACTACATTTTTTGACCCTGCAGGAGGAGGAGACCCAGTTCTATACCAACATCTCTTC

>LCA19_F_R_Tolmomyias_sulphurescens

AGATGGGAACACTATATCTAATTTTTGGTGCCTGAGCCGGAATAGTTGGCACCGCCCTAAGTCTCCTAATCCGAGCAGAACTTGGCCAACCTGGAACCCTCCTAGGAGATGACCAAATCTACAATGTAATCGTTACTGCTCACGCATTCGTAATAATTTTCTTTATAGTAATGCCCATCATAATCGGAGGATTCGGCAACTGATTAGTCCCCCTAATAATTGGAGCACCAGACATAGCATTTCCACGCATGAACAATATAAGCTTTTGACTCCTCCCGCCTTCCTTCCTCCTCCTCCTAGCCTCATCCACGGTAGAAGCCGGAGTTGGCACAGGATGAACTGTCTACCCCCCTTTAGCAGGCAACCTTGCTCATGCTGGAGCCTCTGTAGACCTAGCAATCTTCTCTCTCCACCTAGCGGGTGTTTCTTCAATCCTAGGCGCTATCAACTTCATTACCACAGCAATTAACATAAAACCTCCCGCCCTCTCACAATACCAAACCCCCCTATTCGTATGATCCGTACTAATCACTGCAGTTTTACTCCTTCTCTCCCTACCCGTCCTCGCTGCCGGAATTACCATGCTCCTCACCGATCGCAACCTAAATACTACCTTCTTTGACCCTGCTGGAGGCGGAGATCCAATCCTATATCAACACCTCTTTT

>LCA18_F_R Galbula_ruficauda

AACCAGAAGGAGATGGGCACTCTGTACCTAATTTTCGGGGCTTGGGCCGGCATGATCGGCACTGCCCTTAGCCTCCTCATCCGCGCAGAATTAGGCCAACCAGGCACCCTCCTCGGAGACGACCAAATCTACAATGTAATCGTAACTGCCCACGCCTTCGTCATAATCTTCTTTATAGTCATGCCCATCATAATTGGAGGCTTCGGGAACTGGCTAGTCCCTCTTATAATCGGCGCGCCCGATATAGCATTCCCACGCATAAACAACATAAGTTTCTGACTCCTCCCTCCCTCCTTCCTTCTTCTCCTAGCATCCTCTTCCGTTGAAGCAGGCGCTGGCACAGGATGAACTGTCTACCCCCCACTCGCAGGCAATCTCGCCCATGCTGGTGCTTCAGTAGACCTAGCCATCTTCTCTCTTCACCTTGCAGGGGTCTCCTCAATCCTTGGAGCAATTAACTTTATCACTACCGCTATCAACATAAAACCCCCAGCAATCTCACAATATCAAACCCCCCTATTTGTATGGTCAGTACTAATCACTGCCATCCTACTACTCCTCTCACTACCAGTACTCGCCGCCGGAATTACTATACTCCTAACTGACCGCAACCTAAATACCACCTTCTTTGACCCTGCCGGAGGAGGAGACCCAATCCTCTACCAACACTTATTC

>LCA15_F_R_Ramphocelus_dimidiatus

TCAACCAACCAGAAGGAGATGGGGACTCTGTACCTAATTTTCGGTGCATGAGCCGGAATAGTGGGTACTGCCCTAAGCCTCCTCATTCGAGCAGAACTAGGCCAACCTGGAGCTCTCCTGGGAGACGACCAAGTCTACAACGTGGTCGTCACAGCCCATGCCTTCGTAATAATCTTCTTCATAGTTATGCCAATTATGATCGGGGGATTCGGGAACTGACTAGTCCCATTAATAATTGGAGCCCCAGACATAGCATTCCCACGGATAAACAACATAAGCTTTTGGCTACTTCCCCCATCTTTCCTTCTCCTCCTAGCATCTTCCACAGTCGAAGCAGGGGTCGGTACAGGTTGAACAGTATATCCCCCACTAGCTGGCAACCTAGCCCACGCCGGAGCCTCAGTCGACTTAGCAATCTTCTCCCTACATCTAGCTGGCATCTCTTCAATCCTTGGAGCAATCAACTTCATCACAACAGCAATCAACATGAAACCTCCCGCTCTATCACAATACCAAACCCCCCTATTCGTATGATCAGTATTAATCACTGCAGTCTTACTGCTCCTATCTCTTCCAGTCCTTGCTGCAGGAATTACAATGCTCCTGACGGACCGCAACCTTAACACTACATTCTTCGACCCCGCTGGAGGAGGAGACCCCGTACTATACCAACACCTTTTC

>LCA13_F_R_ Parkesia_novevoracensis

GACCCTATACCTAATTTTCGGCGCATGAGCCGGAATAGTGGGTACCGCCCTAAGCCTCCTCATCCGAGCAGAACTAGGCCAACCCGGAGCTCTTCTGGGAGACGACCAAGTCTACAACGTCGTTGTTACGGCCCATGCCTTCGTAATAATTTTCTTTATAGTTATGCCGATTATAATCGGAGGGTTCGGAAACTGACTAGTCCCCCTAATAATCGGAGCCCCAGACATAGCATTCCCACGAATAAACAACATAAGCTTCTGACTACTACCACCATCATTTCTTCTCCTCCTAGCCTCCTCCACTGTCGAAGCAGGTGTTGGCACAGGCTGAACGGTGTACCCCCCACTAGCTGGCAATCTAGCCCATGCCGGAGCCTCAGTCGACCTAGCAATCTTCTCCCTACACCTGGCCGGTATTTCTTCAATCCTAGGAGCGATTAACTTCATCACAACAGCAATTAACATGAAACCTCCTGCTCTCTCACAATACCAAACCCCCCTATTCGTTTGATCAGTCCTAATCACTGCAGTCCTACTACTCCTGTCTCTCCCAGTCCTAGCCGCAGGAATCACAATGCTTCTCACAGACCGCAMCCTCAACACTACATTCTTCGACCCTGCTGGAGGGGGAGATCCAGTCCTATATCAACATCTCTTC

>LCA12_F_R_Catharus_minimus

ACCCTCTACCTAATTTTCGGCGCGTGAGCCGGAATAGTGGGCACCGCCCTAAGTCTTCTCATCCGAGCAGAACTGGGTCAACCAGGCGCACTACTAGGCGATGACCAGATCTACAACGTAGTTGTCACTGCTCATGCCTTCGTAATAATTTTCTTTATAGTCATGCCAATCATGATTGGAGGGTTCGGAAACTGACTAGTCCCCTTAATAATCGGAGCCCCAGACATAGCATTCCCTCGAATAAACAACATAAGCTTCTGACTTCTTCCACCATCATTCCTCCTCCTCTTAGCCTCCTCCACAGTAGAAGCAGGAGCAGGAACAGGATGAACCGTCTATCCCCCCCTTGCCGGCAACCTAGCACACGCAGGAGCCTCAGTAGACCTGGCTATCTTCTCCCTCCACTTAGCAGGAATCTCCTCAATCCTAGGGGCCATCAACTTCATTACCACAGCTATCAACATAAAACCTCCCGCCCTATCACAATACCAAACCCCCCTATTCGTATGATCAGTACTAATCACTGCAGTCTTACTCCTCCTCTCCCTTCCCGTCCTTGCTGCTGGCATCACCATACTCCTCACCGACCGTAACCTAAACACCACCTTCTTCGACCCAGCAGGAGGAGGAGACCCAGTACTTTACCAGCATCTATTC

>LCA9_F_R_Cantorchilus_leucotis

GCCCAGTTCTCAACCAACCAGAAGGAGATGGGCACCCTATATTTANTCTTCGGCGCATGAGCCGGGATAGTGGGTACCGCCCTGAGCCTCCTCATCCGAGCAGAGCTAGGACAACCCGGCGCTCTACTTGGAGACGACCAAGTCTACAACGTAATTGTCACAGCCCATGCTTTCGTAATAATCTTCTTCATAGTTATACCAATCATAATCGGAGGGTTCGGAAACTGACTAGTCCCCCTAATAATCGGAGCCCCCGACATAGCATTCCCCCGAATAAATAACATAAGCTTCTGACTCCTACCACCATCCTTCCTACTCCTGCTAGCCTCCTCCACAGTCGAAGCGGGAGTCGGAACTGGCTGAACAGTGTACCCTCCCCTAGCAGGCAATCTAGCCCACGCCGGAGCTTCAGTAGACCTTGCTATCTTCTCCCTTCACCTAGCTGGTATTTCTTCCATCCTAGGCGCAATCAACTTTATCACAACAGCAATCAACATAAAACCCCCTGCCCTATCCCAATACCAAACACCCCTATTCGTTTGATCGGTCCTAATCACTGCAGTCCTCCTACTTCTCTCCCTCCCCGTCCTCGCTGCAGGCATCACCATGCTACTGACAGACCGAAACCTAAACACCACCTTCTTCGACCCCGCAGGAGGAGGAGACCCAGTCCTGTATCAACACCTATTC

>LCA8_F_R_Xiphorhynchus_susurrans

ACCAACCAGAAGGAGATGGGCACTCTTTACCTAATCTTCGGAGCATGAGCCGGCATAATTGGAACTGCCCTAAGCCTTCTAATCCGAGCTGAACTCGGACAACCAGGCACCCTTCTAGGTGACGACCAAATTTATAATGTTATCGTCACTGCCCATGCTTTCGTAATAATTTTCTTTATAGTTATACCCATTATAATTGGGGGATTTGGCAACTGATTAGTCCCACTAATAATTGGAGCCCCCGACATAGCATTTCCTCGAATAAACAACATAAGCTTCTGACTCCTCCCCCCATCCTTCCTACTTCTCTTAGCTTCTTCTACAGTAGAAGCAGGTGCAGGAACAGGATGAACAGTCTATCCACCCTTAGCAGGCAATCTAGCTCATGCAGGAGCCTCAGTAGACCTAGCTATTTTCTCCCTTCATCTAGCCGGTGTATCCTCTATCCTAGGGGCTATCAACTTTATTACAACCGCAATCAACATAAAACCACCAGCCCTATCACAATACCAAACTCCCCTATTTGTCTGATCCGTCCTCATCACCGCCGTCCTTCTCCTTCTCTCCCTACCTGTACTAGCTGCTGGTATCACAATGCTCTTAACAGATCGTAACCTAAACACCACATTCTTCGATCCAGCTGGAGGTGGAGATCCCATCCTGTACCAAC

>LCA7_F_R_Momotus_subrufescens

ACCCTATACCTAATCTTTGGCGCATGAGCCGGTATAGCGGGCACCGCCCTAAGTCTACTTATCCGTGCCGAACTAGGCCAACCAGGGACCCTACTAGGAGACGACCAAATTTACAACGTAGTAGTTACTGCCCATGCCTTCGTTATAATTTTCTTTATAGTTATACCAATCATAATTGGCGGATTTGGTAACTGACTAGTTCCCCTCATAATCGGCGCCCCAGACATAGCATTCCCACGTATAAACAACATAAGCTTCTGACTCCTCCCCCCATCCTTCCTACTCCTTCTAGCCTCCTCTACAGTAGAAGCAGGTGCCGGCACCGGATGAACAGTATACCCGCCCCTTGCCGGCAATTTGGCCCACGCAGGAGCCTCAGTAGACTTAGCCATCTTCTCCCTACACCTGGCCGGTGTCTCCTCCATCCTGGGAGCAATCAACTTCATCACAACTGCCACCAACATAAAACCACCCGCCCTCTCACAATACCAAACCCCCCTATTTGTATGATCAGTCCTAATCACCGCCGTGCTACTTCTACTCTCACTCCCTGTTCTAGCCGCTGGTATCACCATGCTATTAACAGACCGTAACCTAAACACTACATTCTTTGATCCGGCTGGGGGAGGAGACCCAGTCCTATACCAACACCTCTTC

>LCA6_F_R_Elaenia_flavogaster

AAAAAGGGGGGCCAGTTCTCAACCAACCAGAAGGAGATGGGTACATTATACCTAATTTTCGGTGCCTGAGCCGGCATAATTGGCACTGCCCTAAGCCTTCTTATCCGAGCAGAACTTGGTCAACCCGGAACCCTCCTAGGAGATGACCAGATCTATAACGTAATTGTTACCGCCCATGCCTTTGTAATAATCTTCTTCATAGTTATACCTATTATAATTGGAGGATTCGGCAACTGACTAGTCCCCCTGATAATTGGAGCCCCTGACATAGCATTCCCACGTATAAATAACATAAGTTTTTGACTCCTTCCCCCATCATTTCTTCTCCTCTTAGCCTCGTCCACAGTTGAAGCCGGGGTAGGAACGGGATGAACCGTTTACCCACCATTAGCCGGCAACCTAGCCCATGCAGGTGCTTCAGTAGACTTAGCTATCTTCTCCCTCCACCTTGCAGGAGTGTCTTCAATCCTAGGTGCTATTAATTTTATTACCACCGCAATCAATATGAAACCACCAGCCCTCTCACAATACCAAACACCTCTATTTGTTTGATCCGTCCTAATTACCGCAGTCCTTCTTCTCCTTTCTCTCCCTGTTCTCGCTGCTGGCATTACCATGCTTCTAACAGACCGAAACCTTAACACTACATTTTTTGACCCTGCAGGAGGAGGAGACCCAGTTCTATACCAACATCTC

>LCA4_F_R_Momotus_subrufescens

TCAACCAACCAGAAGGAGATGGGCACCCTATACCTAATCTTTGGCGCATGAGCCGGTATAGCGGGCACCGCCCTAAGTCTACTTATCCGTGCCGAACTAGGCCAACCAGGGACCCTACTAGGAGACGACCAAATTTACAACGTAGTAGTTACTGCCCATGCCTTCGTTATAATTTTCTTTATAGTTATACCAATCATAATTGGCGGATTTGGTAACTGACTAGTTCCCCTCATAATCGGCGCCCCAGACATAGCATTCCCACGTATAAACAACATAAGCTTCTGACTCCTCCCCCCATCCTTCCTACTCCTTCTAGCCTCCTCTACAGTAGAAGCAGGCGCCGGCACCGGATGAACAGTATACCCGCCCCTTGCCGGCAATTTGGCCCACGCAGGAGCCTCAGTAGACTTAGCCATCTTCTCCCTACACCTGGCCGGTGTCTCCTCCATCCTGGGGGCAATCAACTTCATCACAACTGCCACCAACATAAAACCACCCGCCCTCTCACAATACCAAACCCCCCTATTTGTATGATCAGTCCTAATCACCGCCGTGCTACTTCTACTCTCACTCCCTGTTCTAGCCGCTGGTATCACCATGCTATTAACAGACCGTAACCTAAACACTACATTCTTTGATCCGGCTGGGGGAGGAGACCCAGTCCTATACCAACACCTCTTC

>LCA3_F_R_Catharus_ustulatus

AAAAAAGGGGGCGCCAGTTTCTCAACCAACCAGAAGGAGATGGGTACCCTCTACCTAATTTTCGGCGCATGAGCCGGAATGGTGGGTACTGCCCTAAGCCTTCTCATCCGAGCAGAACTAGGCCAACCAGGCGCACTACTAGGTGACGACCAAATCTACAATGTAGTTGTCACCGCCCATGCCTTCGTAATGATTTTCTTTATAGTTATGCCAATCATGATTGGGGGGTTCGGAAACTGGCTAGTCCCATTAATAATCGGAGCCCCAGACATAGCATTCCCCCGAATAAACAACATAAGCTTCTGACTTCTCCCACCATCATTCCTCCTTCTCCTAGCCTCCTCCACAGTAGAAGCAGGAGCAGGAACAGGATGAACCGTCTATCCACCCCTCGCTGGCAACCTAGCACACGCAGGAGCTTCAGTCGACCTAGCTATTTTCTCCCTACACTTAGCAGGAATCTCCTCAATCCTAGGGGCCATCAATTTCATTACTACAGCAATCAACATAAAACCTCCCGCCCTCTCACAATACCAAACCCCCCTATTCGTGTGATCAGTACTAATCACTGCAGTGCTACTCCTCCTCTCCCTCCCTGTCCTTGCTGCTGGCATCACCATACTTCTCACCGACCGCAACCTAAACACTACCTTCTTCGACCCAGCAGGAGGAGGAGACCCAGTACTTTACCAACACCTCTTC

>LCCA1_F_R_Oryzoborus_funereus

CAGTTCTCAACCAACCAGAAGGAGATGGGANCCNTATACTTAATCTTCGGCGCATGAGCCGGAATAGTAGGTACAGCCCTAAGCCTCCTCATCCGAGCAGAACTAGGTCAACCTGGAGCCCTCCTCGGAGACGACCAAGTCTACAACGTAATCGTCACAGCCCATGCTTTCGTAATAATTTTCTTCATAGTTATGCCGATTATAATCGGAGGATTCGGAAACTGATTAGTCCCCCTAATAATTGGAGCCCCCGATATAGCATTCCCACGAATGAACAATATAAGCTTCTGACTACTTCCCCCATCCTTCCTTCTCCTCCTGGCTTCCTCTACAGTAGAAGCGGGAGTTGGCACAGGTTGAACAGTATACCCCCCATTAGCCGGCAACCTGGCCCACGCCGGAGCCTCAGTCGACCTTGCAATCTTCTCCCTACACCTAGCCGGTATCTCTTCAATCCTAGGGGCAATCAACTTCATTACAACAGCAGTCAACATGAAACCCCCCGCTCTCTCACAATACCAAACCCCCCTATTTGTTTGATCCGTACTAATCACCGCAGTCCTACTACTTTTATCTCTTCCAGTCCTTGCTGCAGGAATCACAATACTCCTTACAGACCGTAACCTTAACACAACATTCTTCGACCCCGCTGGAGGAGGAGACCCCATCCTATATCAACACCTTTTC
